# A Simple Method for Automatic 3D Reconstruction of Coronary Arteries From X-Ray Angiography

**DOI:** 10.3389/fphys.2021.724216

**Published:** 2021-09-07

**Authors:** Minki Hwang, Sa-Bin Hwang, Hyosang Yu, Jaehyeok Kim, Daehyun Kim, Wonjae Hong, Ah-Jin Ryu, Han Yong Cho, Jinlong Zhang, Bon Kwon Koo, Eun Bo Shim

**Affiliations:** ^1^AI Medic Inc., Seoul, South Korea; ^2^Department of Cardiology, School of Medicine, The Second Affiliated Hospital, Zhejiang University, Hangzhou, China; ^3^Department of Cardiology, Seoul National University and Seoul National University Hospital, Seoul, South Korea; ^4^Department of Mechanical and Biomedical Engineering, Kangwon National University, Chuncheon, South Korea

**Keywords:** coronary artery, reconstruction, angiography, deep learning, fractional flow reserve

## Abstract

Automatic three-dimensional (3-D) reconstruction of the coronary arteries (CA) from medical imaging modalities is still a challenging task. In this study, we present a deep learning-based method of automatic identification of the two ends of the vessel from X-ray coronary angiography (XCA). We also present a method of using template models of CA in matching the two-dimensional segmented vessels from two different angles of XCA. For the deep learning network, we used a U-net consisting of an encoder (Resnet) and a decoder. The two ends of the vessel were manually labeled to generate training images. The network was trained with 2,342, 1,907, and 1,523 labeled images for the left anterior descending (LAD), left circumflex (LCX), and right coronary artery (RCA), respectively. For template models of CA, ten reconstructed 3-D models were averaged for each artery. The accuracy of correspondence using template models was compared with that of manual matching. The deep learning network pointed the proximal region (20% of the total length) in 97.7, 97.5, and 96.4% of 315, 201, and 167 test images for LAD, LCX, and RCA, respectively. The success rates in pointing the distal region were 94.9, 89.8, and 94.6%, respectively. The average distances between the projected points from the reconstructed 3-D model to the detector and the points on the segmented vessels were not statistically different between the template and manual matchings. The computed FFR was not significantly different between the two matchings either. Deep learning methodology is feasible in identifying the two ends of the vessel in XCA, and the accuracy of using template models is comparable to that of manual correspondence in matching the segmented vessels from two angles.

## Introduction

Coronary artery (CA) diseases manifested by lumen narrowing is one of the leading causes of death worldwide (Virani et al., 2020). The severity of stenosis is first evaluated by examining the lumen area using medical imaging modalities. However, the coronary blood flow is not necessarily correlated with the visually inspected geometry of the vessel (Park et al., 2012; Toth et al., 2014). One of the methods to determine coronary blood flow is to measure the pressure drops along the stenosis by inserting pressure wire into the vessel (Corcoran et al., 2017). The pressure drop is represented by fractional flow reserve (FFR) which is defined as the ratio of the distal pressure to the proximal pressure through a coronary stenosis (Corcoran et al., 2017). FFR can also be calculated analytically or computationally (Kwon et al., 2014; Lee et al., 2016a, 2017a,b). FFR computation uses reconstructed three-dimensional (3-D) geometry of the vessel, and thus, the accuracy of the 3-D reconstruction is critical to that of the computed FFR.

X-ray coronary angiography (XCA) is among the medical imaging modalities that are used for 3-D reconstruction of the CA. XCA provides relatively clear boundaries of the CA and is widely used in clinical settings. However, XCA provides two-dimensional (2-D) images, and reconstructing 3-D geometry from 2-D images obtained at different angles is not a straightforward task. Back-projection based methods are a group of frequently used methods of reconstruction using 2-D images of XCA (Cimen et al., 2016). In those methods, the locations of 3-D points are determined using the triangulation method (Hartley and Zisserman, 2004) involving the two corresponding points on the two 2-D centerlines obtained at different angles. However, the difficulty lies in establishing correspondence between the two 2-D segmented vessels. Although epipolar constraint (Delaere et al., 1991; Dumay et al., 1994) is generally used for correspondence, it often does not generate a single match (Zhang et al., 1995; Cong et al., 2015). Vessel overlapping and foreshortening, which is shortened 2-D image of the vessel due to the relative orientation of the vessel to the imaging device, often occur in 2-D images of XCA.

In this study, we present a method of automatically establishing correspondences among the centerline points on the 2-D images obtained at two different angles in XCA, especially focusing on overcoming the problem of foreshortening. The two ends of the vessel were identified using a deep learning methodology. The correspondences between the centerline points on the two 2-D segmented vessels were established using template models. The reconstructed 3-D models were compared with those obtained by manual matching.

## Materials and Methods

### Angiography Image Data Acquisition

The angiography image data were retrospectively obtained from the patients who underwent XCA in Seoul National University Hospital between January 2015 and October 2019. XCA was performed using standard techniques. Angiographic views were obtained after administration of intracoronary nitrate (100 or 200 μg).

### Identification of the Two Ends of the Vessel

**FIGURE 1:**
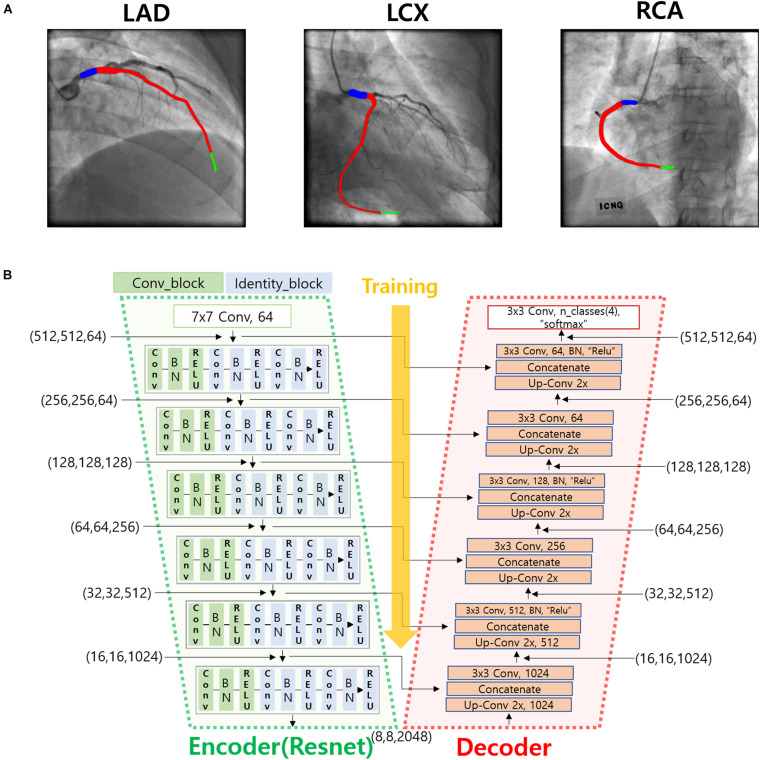
Deep learning method for the identification of the two ends of the vessel. **(A)** An example of the labeling of the two ends (blue and green) and the vessel inside (red) for LAD, LCX, and RCA. **(B)** Deep learning network used for the training of the labeled images.

**FIGURE 2:**
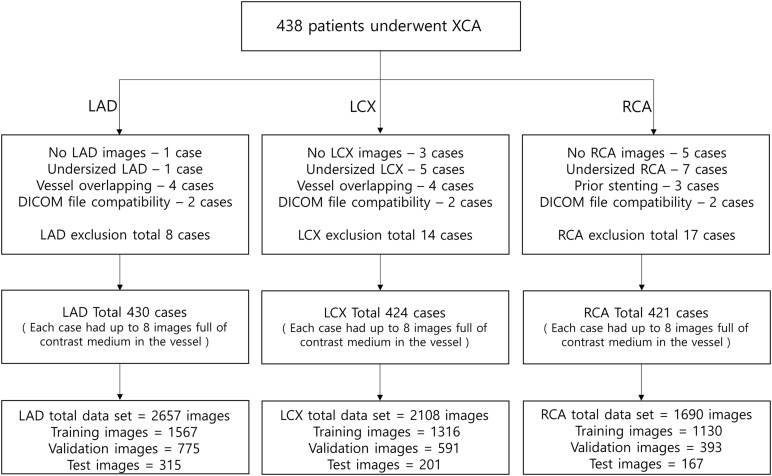
The number of images used for the deep learning network. The cases were divided into the training, validation, and test cases randomly with the ratio of 6.3:2.7:1, which resulted in the number of images shown in the bottom boxes. Each case had up to 8 images full of contrast medium in the vessel.

To automatically identify the two ends of the vessel in each of the two images obtained at different angles, a deep learning method was adopted. Training images for the deep learning network were created by labeling the two ends of the vessel on the angiogram images (Figure 1A). The proximal area of the vessel was labeled at the tip of the catheter. The distal area of the vessel was labeled at the distal end of the contrast material on the images when the material fully filled the vessel. For the deep learning network, we used a U-net consisting of an encoder (Resnet) and a decoder (Figure 1B). The network was trained with 2,342, 1,907, and 1,523 labeled images for the left anterior descending (LAD), left circumflex (LCX), and right coronary artery (RCA) images, respectively. The network was trained using Nadam optimizer (Dozat, 2016) with learning rate of 0.0001. Batch size of 4 and 150 epochs were implemented. The variables were initialized with random numbers between 0 and 1 with uniform distribution. Figure 2 summarizes the number of images used. The cases were divided into the training, validation, and test cases randomly with the ratio of 6.3:2.7:1, which resulted in the number of images shown in Figure 2.

### Two-Dimensional Segmentation of the Vessel

Two-dimensional angiogram images were segmented by applying Frangi filter (Frangi et al., 1998) to enhance the area where contrast material exits. To determine the centerline of the vessel, a fast-marching algorithm was used to find the fastest path from the start point to the end point with a velocity weight proportional to the distance to the nearest wall (Van Uitert and Bitter, 2007). The diameter of the vessel was obtained by averaging the distances from the centerline point to the two borders in the direction normal to the centerline at each centerline point. These diameters measured on the 2-D images were later converted to those in 3-D space when 3-D reconstruction was performed.

### Establishment of the Correspondence Using Template Model

**FIGURE 3:**
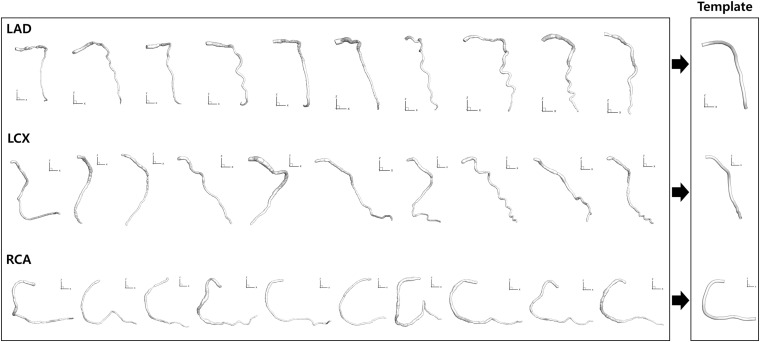
Template model generation. Ten models for each CA reconstructed from a previous study (Lee et al., 2016b) were averaged to generate template models.

To overcome the problem of foreshortening in angiography images when establishing the correspondence between the two 2-D segmented vessels from two different angles, template models of the centerlines of the CAs were used. Each template model of the centerlines of LAD, LCX, and RCA was constructed by averaging the centerline points of 10 randomly selected models from a previous study (Lee et al., 2016b), which were generated by using a commercial software (AutoSeg, Ver. 1.0, AI Medic Inc., Seoul, South Korea) (Figure 3). The 10 models were resized so that the lengths of the centerlines were the same. The 10 models were also translated so that the start points were identical. One hundred equidistantly distributed centerline points were generated from the start to the end locations for each of the 10 models, and the coordinates of the centerline points were averaged among the 10 models (Figure 3).

**FIGURE 4:**
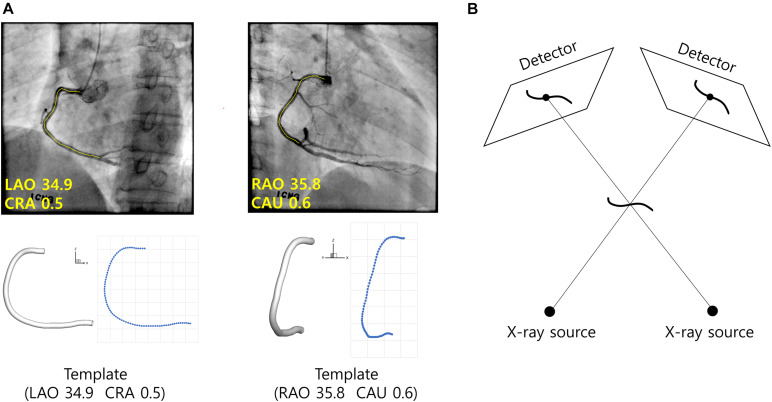
Reconstruction of CA using template model. **(A)** Template model is viewed from the same angles the angiograms were obtained. For each centerline point of the template model, the distance from the start point along the centerline is calculated and the ratio to the total length is calculated. The correspondences between the segmented vessel centerlines (yellow) are established using the distance ratios obtained from the template model. **(B)** Geometrical relationships among the CA in 3-D space, X-ray sources and the vessel images on the detectors.

For the given set of angiograms, two-dimensional coordinates of the template centerline points were calculated when viewed from the same angles at which the given angiograms were obtained in terms of left/right anterior oblique (LAO/RAO) and cranial/caudal (CRA/CAU) angles (Figure 4A). For each centerline point, the distance from the start point along the centerline was calculated at the 2-D plane, and the ratio to the total distance from the start to the end point was also calculated. The distance ratios were used to determine the matching points on the two 2-D centerlines in the actual angiograms (Figure 4A).

### Three-Dimensional Reconstruction of the Vessel

The 3-D coordinates of the centerline points were determined in a point-by-point manner using a back-projection based method (Cimen et al., 2016). Briefly, each centerline point in the 3-D space was determined using geometrical relationships among the locations of X-ray sources at the two angles, and the coordinates of the points on the 2-D images at the two angles (Figure 4B). First, a line is formed between the X-ray source and a centerline point on the 2-D image at the first angle. The 3-D point is searched on the line such that the projection to the 2-D image at the second angle is closest to the corresponding point on the image. Patient table panning was incorporated in the process by adding two more search parameters defining the movement of the 3-D point on the plane parallel to the table. The vessel diameters measured on the 2-D images in the segmentation step were converted to 3-D diameters after the coordinates of the 3-D points were determined.

### Computation of FFR

FFR was obtained using a commercial software (HeartMedi, Ver. 1.0, AI Medic Inc., Seoul, South Korea). Briefly, unstructured tetrahedral meshes were created inside the 3-D vessel geometries for computation. Incompressible Navier-Stokes equations were solved numerically. Blood flow velocity obtained from angiography frame count analysis was converted to hyperemic velocity using the formula used in Carson et al. (2019). The hyperemic velocity was used as the inlet boundary condition for the computation. Steady state simulation was performed, and the pressures obtained from the simulation was used to calculate P_*d*_/P_*a*_ where P_*d*_ and P_*a*_ are distal and inlet pressures, respectively.

## Results

### Identification of the Two Ends of the Vessel

**FIGURE 5:**
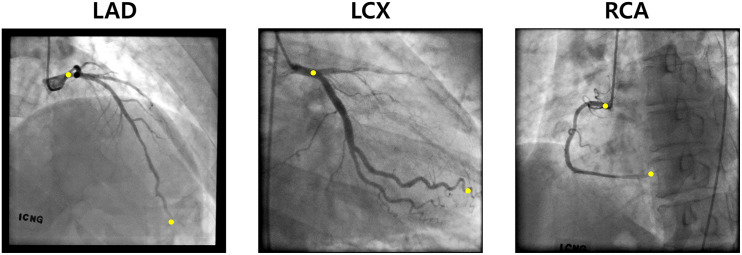
Examples of the two ends (yellow) pointed by the deep learning network for LAD, LCX, and RCA.

Figure 5 shows an example of the start and end points of the LAD, LCX, and RCA indicated by the trained deep learning network. The total success rates of the indication of the start and end points were 97.3 and 93.3%, respectively, among 683 test images for each end. The indication was regarded as successful when the indicated point was located on the vessel within the 20% of the total vessel length from either the actual start or end point. The success rates for the start point in the cases of LAD, LCX, and RCA were 97.7, 97.5, and 96.4%, respectively, and for the end point, they were 94.9, 89.8, and 94.6% among 315, 201, and 167 test images, respectively. The success rates for pointing the vessel area within 5, 10, 15, and 20% of the total vessel length from the two ends are shown in Table 1. The test image acquisition angles were (RAO 30.5 ± 2.12, CRA 34.1 ± 4.58), (RAO 23.0 ± 8.43, CAU 29.1 ± 5.82), and (LAO 36.5 ± 2.07, CRA 20.0 ± 11.4) for LAD, LCX, and RCA, respectively, for the first views, and for the second views, they were (LAO 38.6 ± 2.84, CRA 23.7 ± 4.45), (RAO 1.91 ± 0.94, CAU 35.4 ± 7.89), and (LAO 36.1 ± 1.91, CAU 0.04 ± 0.69) for LAD, LCX, and RCA, respectively.

**TABLE 1:** Success rates of pointing the vessel ends by the deep learning network.

Defined length of vessel end with respect to total vessel length (%)	Vessel type	Success rate (%)	
		
		Start	End
	LAD	97.7	94.9
20	LCX	97.5	89.8
	RCA	96.4	94.6
	LAD	96.8	90.4
15	LCX	97.5	83.5
	RCA	96.4	87.3
	LAD	92.3	82.2
10	LCX	93.5	69.1
	RCA	86.7	70.4
	LAD	88.2	63.8
5	LCX	92.5	62.1
	RCA	83.1	54.2

### Establishment of the Correspondence Using Template Model

The template models averaged among 10 randomly selected models for LAD, LCX, and RCA are shown in Figure 3. When all the 10 models and the template model were translated so that the start points were identical, the distances among the models increased as the distance from the start point increased along the centerline. For LAD, the average distance from the template model to each random model was 0.20 ± 0.07 at the end point when the centerline length from the start to the end point was 1. For LCX and RCA, they were 0.23 ± 0.09 and 0.15 ± 0.07, respectively.

**FIGURE 6:**
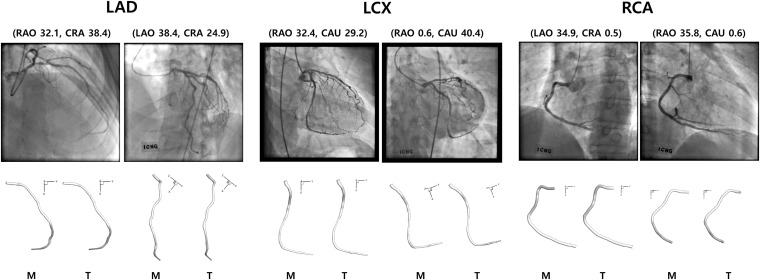
Reconstructed 3-D models for LAD, LCX, and RCA using manual (M) and template (T) matchings.

Because the 3-D centerline points were determined among the 3-D points on the line connecting the X-ray source and the points on the 2-D image at the first angle, the accuracy of the reconstructed 3-D centerline were examined by comparing the projected and segmented 2-D centerlines at the second angle. When the template models were used in the matching of the centerline points between the two images from two angles, the average distance between the projected and segmented centerline points at the second angle (ADPS2) was 1.66 ± 1.56 pixels for a model of LAD. The total number of pixels in each image was 512 by 512. The 3-D model was also created by manually matching four landmark points on the centerlines of 2-D images for comparison. In the case of the manual matching, the ADPS2 was 1.35 ± 0.74 pixels (Figure 6). There was no statistically significant difference between the ADPS2s for the template and manual matchings based on an independent samples *t*-test (*p* = 0.11, *n* = 100). For a model of LCX, ADPS2s were 1.76 ± 0.94 and 1.55 ± 0.81 (*p* = 0.09, *n* = 100) for template and manual matchings, respectively. For a model of RCA, they were 1.48 ± 1.04 and 1.59 ± 1.09 (*p* = 0.49, *n* = 100), respectively. We also generated template models using 5 and 15 coronary models for each artery, and the ADPS2s were not significantly different between the template and manual matchings for all the three types of the artery when 15 coronary models were used for template model generation (Supplementary Material). However, when 5 models were used for template model generation, the ADPS2s were significantly different between the template and manual matchings for all the three types of the artery (Supplementary Material).

### Computation of FFR

**FIGURE 7:**
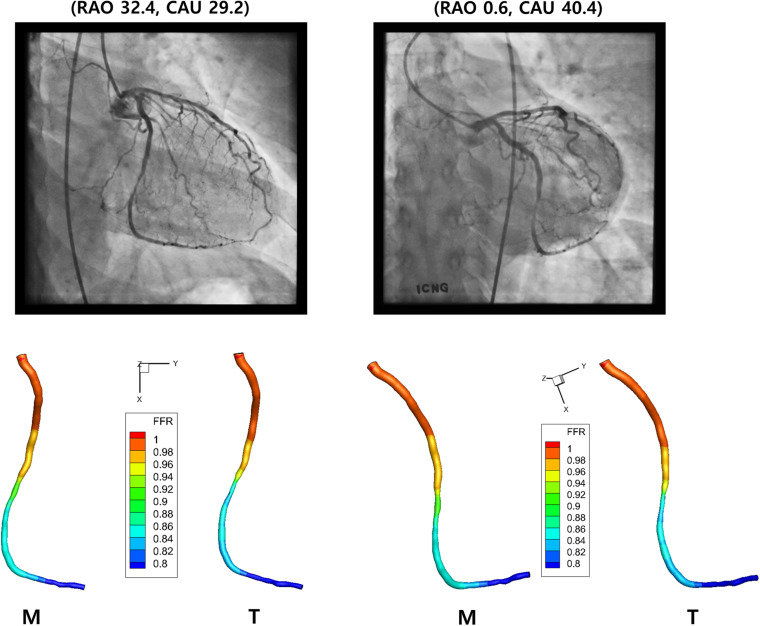
Computed FFR. Distribution of computed FFR is shown for LCX for two different views. Manual (M) and template (T) matchings are compared.

The values of FFR were also compared between the 3-D models created by the template and manual matchings (Figure 7). Table 2 shows the FFR values at the locations of 25, 50, and 75% of the total length from the inlet along the centerline for LAD, LCX, and RCA. The largest difference of FFR values between the two matching methods was 2.3% observed in the case of LCX at 75% location, which seems to be due to the mild stenosis located in the middle part of the vessel. All the other cases showed less than 1% of the difference of FFR values between the two matching methods.

**TABLE 2:** Computed FFR for manual (M) and template (T) matchings.

Distance from inlet (%)	LAD		LCX		RCA	
			
	M	T	M	T	M	T
25	0.965	0.960	0.987	0.988	0.961	0.963
50	0.927	0.921	0.916	0.911	0.926	0.929
75	0.879	0.872	0.844	0.825	0.895	0.898

## Discussion

Automatic 3-D reconstruction of the CAs using medical images is still a challenging task. Currently available imaging modalities have their own limitations in resolution and the appropriate interpretations of the resulting images require significant amount of the knowledge in the working of the imaging device and the anatomy of the arteries. Although XCA provides images of relatively high resolution, the automatic matching of the centerlines obtained at two different angles needs to be established for automatic 3-D reconstruction. This study tested the possibility of utilizing deep learning and template models of the arteries in achieving that goal.

One of the commonly used methods of establishing correspondence between the centerlines obtained at two different angles is utilizing epipolar lines resulting from the epipolar planes defined by the object points and the two X-ray sources (Delaere et al., 1991; Dumay et al., 1994; Chen and Carroll, 2000, 2003). Although it is a theoretically accurate method of establishing correspondences, uncertainties such as patient table panning between the two acquisitions from different angles move the corresponding points away from the epipolar lines in the second view image. Although the degree of the uncertainties can be estimated using optimization processes (Chen and Carroll, 2000, 2003; Yang et al., 2009), the search of the accurate corresponding points should be included in the optimization process (Blondel et al., 2006), which would make the whole process complicated and time-consuming. In addition, a part of the vessel could be parallel with the epipolar lines, which also would complicate the determination of the corresponding points (Yang et al., 2014; Cong et al., 2015). Bifurcation points of the arteries can be landmarks in the matching of the vessel trees obtained from two angles (Andriotis et al., 2008; Galassi et al., 2018). However, automatic detection of all the branches is not always feasible because some branches are often indistinguishable in the angiographic images. Moreover, some curved branches sometimes overlap with the main vessel, which makes the distal end of the branch seem like a new branch.

For fully automatic 3-D reconstruction of the CA using XCA, automatic determination of the two ends of the vessel on 2-D images is an important step (Vukicevic et al., 2018). Those ends selected from the images obtained from two different angles should be correspondent. The methodology of deep learning turned out to be useful in the selection of the end points in this study. The selection of the proximal end was relatively more successful compared to that of the distal end, because the tip of the catheter was relatively clear for deep learning network to identify. For the identification of the distal end, it was helpful that the viewing angles the clinicians prefer were more or less set for each CA (Yang et al., 2019). As a result, the distal ends were located more or less within a certain area in the 2-D image of XCA for each CA. The difference of the locations of the proximal ends on the two images from different angles would also provide a clue for the degree of the table panning and cardiac/respiratory motions. The accuracy of the identification remains to be improved for fully automatic reconstruction.

Besides the automatic establishment of correspondences between the centerline points and the determination of the two ends of the vessel focused on in this study, fully automatic 3-D reconstruction of the CA from XCA requires more steps such as the determination of the shape of the cross-section of the vessel (Vukicevic et al., 2018) and the compensation of the cardiac/respiratory motions (Chen and Carroll, 2003; Blondel et al., 2006; Zheng and Qi, 2011). Also, for the computation of FFR, another advantage of XCA is that the blood flow velocity can be extracted by analyzing the time-dependent locations of the contrast medium (Kunadian et al., 2009). The inclusion of all these components would enable more accurate 3-D reconstruction of the CA and help diagnosis of CA disease. Although the reconstructions using template models were comparable to those using manual matchings in this study, validations against synthetic phantoms or catheter-based FFR measurements need to be performed to evaluate the accuracy of the present approach. Especially, the FFR simulations need to be performed for vessels with complex stenotic lesions to fully validate the present reconstruction method. Also, the present method needs to be compared with pre-existing 3-D reconstruction methods. It is important to know the uncertainty or confidence in the predictions from computational tools. Although the comparison of ADPS2 between the template and manual matchings for using 5, 10, and 15 coronary models for template model generation could provide a rough idea of the uncertainty in the predictions from the present method, we believe a more rigorous methodology should be designed and implemented to know the confidence in the predictions. Although the methods presented in this study are approximate approaches, they could be combined with currently available methods and provide ideas for future development of more rigorous ones.

## Data Availability Statement

The datasets generated for this study are available on request to the corresponding author.

## Ethics Statement

The studies involving human participants were reviewed and approved by The Institutional Review Board of the Seoul National University Hospital. The patients/participants provided their written informed consent to participate in this study.

## Author Contributions

ES, BK, and HC provided the main idea for this research and edited the manuscript. JZ obtained the data. MH, S-BH, HY, JK, DK, WH, and A-JR analyzed the data. MH wrote the initial draft of the manuscript. All authors reviewed the manuscript.

## Conflict of Interest

MH, S-BH, HY, JK, DK, WH, A-JR, HC, and ES were employed by the company AI Medic, Inc. The remaining authors declare that the research was conducted in the absence of any commercial or financial relationships that could be construed as a potential conflict of interest.

## Publisher’s Note

All claims expressed in this article are solely those of the authors and do not necessarily represent those of their affiliated organizations, or those of the publisher, the editors and the reviewers. Any product that may be evaluated in this article, or claim that may be made by its manufacturer, is not guaranteed or endorsed by the publisher.
